# A Data-Gathering Scheme with Joint Routing and Compressive Sensing Based on Modified Diffusion Wavelets in Wireless Sensor Networks

**DOI:** 10.3390/s18030724

**Published:** 2018-02-28

**Authors:** Xiangping Gu, Xiaofeng Zhou, Yanjing Sun

**Affiliations:** 1 College of Computer and Information, Hohai University, Naijing 210098, China; xpgu2010@hyit.edu.cn; 2 Faculty of Electronic Information Engineering, Huaiyin Institute of Technology, Huai’an 223003, China; 3 School of Information and Control Engineering, China University of Mining and Technology, Xuzhou 221116, China; yjsun@cumt.edu.cn

**Keywords:** compressive sensing, wireless sensor networks (WSNs), diffusion wavelets, ant colony algorithm, data gathering

## Abstract

Compressive sensing (CS)-based data gathering is a promising method to reduce energy consumption in wireless sensor networks (WSNs). Traditional CS-based data-gathering approaches require a large number of sensor nodes to participate in each CS measurement task, resulting in high energy consumption, and do not guarantee load balance. In this paper, we propose a sparser analysis that depends on modified diffusion wavelets, which exploit sensor readings’ spatial correlation in WSNs. In particular, a novel data-gathering scheme with joint routing and CS is presented. A modified ant colony algorithm is adopted, where next hop node selection takes a node’s residual energy and path length into consideration simultaneously. Moreover, in order to speed up the coverage rate and avoid the local optimal of the algorithm, an improved pheromone impact factor is put forward. More importantly, theoretical proof is given that the equivalent sensing matrix generated can satisfy the restricted isometric property (RIP). The simulation results demonstrate that the modified diffusion wavelets’ sparsity affects the sensor signal and has better reconstruction performance than DFT. Furthermore, our data gathering with joint routing and CS can dramatically reduce the energy consumption of WSNs, balance the load, and prolong the network lifetime in comparison to state-of-the-art CS-based methods.

## 1. Introduction

Wireless Sensor Networks (WSNs) generally consist of a large number of sensor nodes and a sink node deployed in the detected environment to monitor various physical characteristics of the real world, such as temperature, voltage, wind direction, and so on. Furthermore, WSNs should have a long enough lifetime to successfully fulfill the monitoring task. However, sensor nodes are limited in terms of computational ability, communication bandwidth, and energy availability. In many cases, such as where there is an inaccessible or hostile field, a battery is difficult to recharge [1]. Therefore, how to gather sensor network data in an energy-efficient way becomes the crucial problem in practical applications. For instance, [2] indicates the importance of promoting the efficiency of intra-car multihop WSNs.

A great many research works have addressed energy-efficient challenges for WSNs from the point of view of sleep schedule [3], topology control [4], area coverage [5], mobile sink [6], and data gathering [7]. However, the core of the study is to take advantage of data-gathering and routing algorithms in WSNs to promote network performance and balanced load.

We can leverage the spatial characteristics in sensor node readings from real deployments, which is an essential technique to decrease data transmission costs while preserving relatively high recovery accuracy in the sink node [1]. In other words, the spatial correlation property of a sensor node leads to inherent data sparsity in some areas, such as wavelet domain and DCT domain. In order to solve the sparsity of such signals, compressive sensing (CS) is exploited as a novel signal-processing paradigm that provides an efficient compressive method and recovers sparse or compressible signals [8,9,10,11].

In recent years, a large number of CS-based techniques have been investigated to gather data on WSNs [12,13,14,15,16,17,18,19]. However, most of the current CS-based methods neglect the topology structure or property of sensor node readings, such as spatial characteristics. Therefore, the performance of the algorithm is limited. In this paper, the spatial property of sensor node readings is exploited to strengthen the performance of networks, considering the topology structure and sensor nodes’ distance. Thus, we take advantage of spatial correlations of sensor node readings to further promote the efficiency of the data-gathering algorithm.

So far, various CS-based data-gathering schemes have been presented to promote the networks’ efficiency for data gathering in WSNs [12,13,14,15]. (See Section 2 for more details.) For instance, [12] proposes a data-gathering scheme that diminishes the bottleneck of the sink in WSNs. In [13], the spatial correlation of sensor node readings is leveraged to improve the performance of WSNs. Wu et al. propose a sparsest representation basis, which makes full use of the spatial property of sensor node readings [14]. Quer et al. introduce a framework for data gathering considering CS and principal component analysis (PCA) simultaneously. In this scheme, the spatial characteristics of sensor node readings are utilized to enhance the recovery accuracy [15].

The routing protocol in data-gathering schemes has also attracted much attention in the past few years [16,17,18,19]. In [16], multi-chain CS-based data gathering is presented by considering the routing protocol from the sensor nodes to the sink of WSNs. A special routing algorithm corresponding to the topology structure is given in [17]. In [18], a random walk routing approach is introduced to reduce the energy consumption of the whole network. In [19], Xie et al. propose a cluster-based routing algorithm for data gathering in WSNs. However, these approaches do not consider the property of sensor node readings. Additionally, the optimal routing algorithm is not considered.

In this paper, a sparser basis based on improved diffusion wavelets is provided, aiming to exploit the sensor node’s spatial correlation features. Moreover, a novel data-gathering algorithm with joint CS and modified ant colony routing is presented. More specifically, the contributions of this paper are as follows.

- Improved diffusion wavelets are used as the basis for data gathering, which makes full use of sensor nodes’ spatial correlations;
- A sparse measurement matrix is used, where the non-zero components of each row denote the offspring nodes of one projection node, which only requires a fraction of nodes to participate in each measurement task, leading to a dramatic decrease in the energy consumption.
- Modified ant colony routing compressively considering the next hop sensor node’s residual energy and path length is proposed; to speed up the algorithm coverage ratio and avoid a local optimal, the pheromone impact factor is improved. Furthermore, a novel data-gathering scheme that integrates MST (Minimum Spanning Tree), modified ant colony routing, and compressive sensing is provided;
- Theoretical proof is shown on the equivalent measurement matrix to satisfy the RIP.
- The superiority of our approach is demonstrated by numerical experiments with synthetic and real sensor data. The simulations show that our sparse basis sparsity the signal. In addition, our approach can accurately reconstruct the original signal, thereby reducing the energy consumption of WSNs and balancing the network load.

The reminder of this paper is organized as follows. First, in Section 2, related work is introduced. Section 3 discusses preliminaries on CS theory and the system model. Section 4 analyzes a lot of WSNs datasets and signal spatial correlation characteristics. Furthermore, the proper sparse basis is designed using a modified diffusion wavelet. Moreover, it provides the modified ant colony routing algorithm. In addition, we describe a novel compressive data-gathering algorithm with joint improved ant colony algorithm and MST. Section 5 proves that the equivalent measurement matrix satisfies the RIP. The numerical results of synthetic and real datasets are analyzed in Section 6. Conclusions and future work are given in Section 7.

## 2. Related Work

Recently, numerous CS-based data-gathering approaches have been provided to increase the network’s lifetime by decreasing the amount of transmission data and balancing load of the whole WSNs [12,13,20,21]. Luo et al. first analyzed the CS-based data-gathering scheme (plain CS) in large-scale WSNs, namely, compressive data gathering (CDG) [12]. In [12], Luo et al. prove that the network capacity is proportional to the sparsity level of sensor node readings. However, this method also demonstrates that an increase in the number of measured samples leads to growth of transmission costs compared to the non-CS approach. CStorage [13] exploits the spatial correlation of sensor node readings along with CS to reduce the number of transmission data points, aiming at prolonging the WSNs’ lifetime. Bajwa et al. introduce compressive wireless sensing, whereby a distributed, matched-source channel communication architecture was proposed for energy-efficient estimation of sensor node readings [20]. However, sensor node readings’ spatial correlation features in [20] are not considered. The authors of [21] show that hybrid CS can achieve high network throughput, while plain CS may not yield a significant improvement in throughput because of the dense measurement matrix. 

Up to now, the interaction between routing and compression data gathering has seriously hindered the progress of CS in WSNs [12,22]. These data-gathering methods jointly use routing and CS to mitigate the data throughput. The authors of [22] address the data collection problem in WSNs, with routing used in combination with CS to transmit random data projections. However, this technique demonstrates that a sparse measurement matrix not only deviates from RIP property, but does not minimize transport energy consumption for each CS measurement. Nevertheless, in [12], Luo et al. claim that measurement matrix [I R] has good RIP; in addition, CDG can further reduce communication costs for both chain-type and tree-type routing. Alternatively, this method requires a large number of sensor nodes involved in each projection gathering, bringing about high transmission costs. To significantly reduce data traffic and save power, distributed multi-chain compressive sensing based on a routing algorithm in WSNs is presented. However, the measurement matrix is still a dense measurement matrix [16]. In [23], a distributed sparse random measurement is proposed, where the sparse or compressible signal can be recovered. Wang et al. consider that some sensor nodes instead of all of them are required in each measurement. Furthermore, the authors of [23] show that sparse random projection significantly enhances network performance. The communication costs can be reduced to Ο(logN) packets per sensor, but this strategy utilizes random routing in the networks, which mitigates the energy efficiency of WSNs. In [14] a sparest random scheduling for compressive data gathering in WSNs is provided to satisfy RIP property; a sparse basis is designed based on the measurement matrix and sensor node readings. The scheme claims that it can achieve good results in real datasets, but the routing is not considered. Routing and CS are also incorporated into [17], which considers special random routing of a given network topology. However, sensor nodes’ compressible ration and routing schemes considerably affect the transport costs. In fact, this method is impractical since sensor nodes are randomly deployed in the field of WSNs applications. In [18], Zheng et al. introduces an algorithm based on a random walk that is independent of network topology. Nevertheless, those techniques in [14,17,18,23] suffer from the fact that the routing of the projection to the sink node is not optimal, which leads to an extra transmission bottleneck for the sensor nodes near the sink node in WSNs. [19] follows the dense matrix used in [12] and hybrid CS in [21]; a cluster-based data-gathering scheme is proposed. This method indicates that it reduces the transport cost and balances the traffic load of the network, but the same issue of a dense matrix exists in [19]. Furthermore, there is no theoretical proof that the equivalent measurement matrix satisfies the RIP in [19].

Existing CS-based data-gathering algorithms adopt random, chain-type and tree-type routing in which numerous sensor nodes participate in each measurement. However, few studies take into account optimal routing in CS-based data gathering. Moreover, design the sparse basis does not make full use of the spatial correlation of the sensor nodes and network topology. In addition, there is no theoretical guarantee that the equivalent sensing matrix obeys RIP in most research works.

## 3. Preliminaries

### 3.1. CS Theory

CS is a novel technique that is used to compress and recover an original signal when it has sparse representation in some domains. As above, we consider the sparse signal $X\in {\mathbb{R}}^{N}$, describing the sensor node readings in WSNs with N nodes. Χ is represented as follows:

(1) $$X=\Psi S=\sum_{i=1}^{N}\psi_{i}s_{i},$$

where $\Psi =[\psi_{1},\psi_{2},\ldots ,\psi_{N}]\in {\mathbb{R}}^{N\times N}$ is some basis matrix, and $S\in {\mathbb{R}}^{N}$ is coefficient vector. When Χ can be represented as a linear combination of K column vectors of Ψ with K≪N, it means that the signal is K-sparse, which demonstrates that it has only K nonzero components or (N-K) smallest components can be ignored. In this case, Ψ is referred to as the sparse basis. Thus, the information can be compressed by simple linear projection. This projection matrix $\Phi \in {\mathbb{R}}^{M\times N}$ is an M×N measurement matrix, $Y\in {\mathbb{R}}^{N}$ is the measurement vector, and K<M<N. The compressed measurements can be described as

(2) Y=Φx=ΦΨS=AS,

where A denotes the equivalent sensing matrix.

The measurement matrix needs to satisfy the RIP conditions [24].

**Definition** **1.** *(RIP [10,11]): A matrix* Φ *satisfies the restricted isometric property of order* K *if there exists a* $\delta_{K}\in (0,1)$ *such that*

(3) $$(1-\delta_{K}){\left\|x\right\|}_{2}^{2}\leq {\left\|\Phi x\right\|}_{2}^{2}\leq (1+\delta_{K}){\left\|x\right\|}_{2}^{2}$$

*for all* K*-sparse vectors* $S\in {\mathbb{R}}^{N}$.

However, the reconstruction of the original signal x from y is a problem. Candès and Tao [9] and Donoho [10] have shown that signal X can be reconstructed via $\ell_{1}$ as

(4) $$\overset{\wedge }{x}=\underset{x}{\arg\min}{\left\|x\right\|}_{1}\;s.t.\;Y=\Phi X,$$

where Φ satisfies RIP and M≥O(K·log(N/K)), X can be recovered successfully with high probability. In addition, there are a large number of reconstruction algorithms such as the basis pursuit (BP) algorithm [24], orthogonal matching pursuit (OMP) algorithm [25], CosaMP [26], Stage-wise Orthogonal Matching Pursuit (StOMP) [27], gOMP [28], and so on.

### 3.2. System Model

We describe WSNs where N sensor nodes are randomly deployed in a square area. The system model is represented by a connected graph G(V,E), where the vertex set V denotes the nodes in the networks, and the edge set E denotes the wireless links between the different nodes. Node i can communicate with node j if they are involved in the communication range. We assume that the single hop distance $d_{i}{}_{j}$ between node i and node j can be represented as a Euclidean distance. At a sampling instant, each sensor node i takes a measurement $x_{i}$; the goal of the data gathering in WSNs is to collect sufficient information to reconstruct the N-dimensional signal $X={\left[x_{1},\ldots ,x_{N}\right]}^{T}$. In this paper, the energy consumption model is similar to that in [29], namely, Equation (5) and Equation (6). When the distance between transmission node i and receive node j is greater than $d_{0}$, the multi-path fading model is utilized. When the distance is less than $d_{0}$, the free-space model is adopted.

(5) $$E_{Ti}(L,d)=\left\{ \begin{array}{ll} E_{elec}\times L+E_{amp}\times L\times d^{4}, & d\geq d_{0}\\ E_{elec}\times L+E_{fs}\times L\times d^{2}, & d<d_{0} \end{array}\right.$$

(6) $$E_{R}{}_{j}(L)=E_{elec}\times L,$$

where $E_{Ti}(L,d)$ and $E_{R}{}_{j}(L)$ describe the energy consumption of transporting and receiving the L bit data packet. $E_{elec}$ denotes the power expended to run the transmitter or receiver circuitry of the sensor node. $E_{amp}$ and $E_{f}{}_{s}$ represent energy consumption for a multi-path fading amplifier and free-space amplifier, respectively.

## 4. Our Proposed Algorithm

### 4.1. Datasets

The spatial correlation features among the different sensor nodes, which can be exploited to considerably reduce transmission costs in WSNs in [15] by analyzing the signal features utilizing PCA technique. However, in this paper, we follow the information theory as in [30] to evaluate spatial correlation characteristics of real sensor nodes’ data in WSNs. The entropy concept in information theory denotes the information involved in datasets. We investigate the properties of the signal in view of the spatial marginal and conditional entropy because the gap between marginal entropy and conditional entropy demonstrates data compressibility. We extract five different matrixes from DEI [31], IntelLab [32], LUCE-EPFL [33], CitySense [34], and OrangeLab [35], which are deployed in campus, indoor, and urban city environments. These data features are summarized in Table 1.

Let $x_{i,t}$ represents the i sensor node readings at slot t. The signal can also be expressed using matrix-type Equation (7), where the rows are the ith sensor node readings and the columns are sensor node readings at t slot.

(7) $$X=\left[\begin{array}{lllll} x_{0,0} & \ldots & x_{0,t} & \ldots & x_{0,T-1}\\ x_{1,0} & \ldots & x_{1,t} & \ldots & x_{1,T-1}\\ \ldots & \ldots & \ldots & \ldots & \ldots \\ x_{N-1} & \ldots & x_{N-1,t} & \ldots & x_{N-1,T-1} \end{array}\right]$$

#### 4.1.1. Spatial Marginal and Conditional Entropy

Sensor node readings are different from the signals of a 2D image, which has a discrete sequence range from 0 to 255, while the signal collected from WSNs is continuous. Therefore, we need to preprocess the signals before calculating spatial marginal and conditional entropy. Take temperature sequence [24.23 24.66 24.75 23.65 23.70 24.12 22.78 22.23 22.16 22.96 21.29 21.11] for example: the data can be divided into Q equal sections (select proper equal sections depending on the real data; here, Q is 4); we calculate the occurrence probability per section so that the marginal entropy yield is 1.9591 bits.

The spatial marginal entropy is defined as the entropy of different nodes sensing the state at slot t. Suppose $\alpha_{k}$ is the occurrence frequency of state $z_{k}$ in the t column $Z_{t}$. The probability of $P(z_{k})$, spatial marginal entropy $H(Z_{t})$, and conditional entropy $H(Z_{i,t}|Z_{j,t})$ are expressed by Equations (8)–(10) respectively:

(8) $$P(z_{k})=\underset{N\to \infty }{\lim}\frac{\alpha_{k}}{N}$$

(9) $$H(Z_{t})=-\sum_{t=0}^{Q-1}P(z_{t})\cdot \log_{2}P(z_{t})$$

(10) $$H(Z_{i,t}|Z_{j,t})=H(Z_{i,t},Z_{j,t})-H(Z_{j,t}).$$

#### 4.1.2. Spatial Compressibility

**Figure 1:**
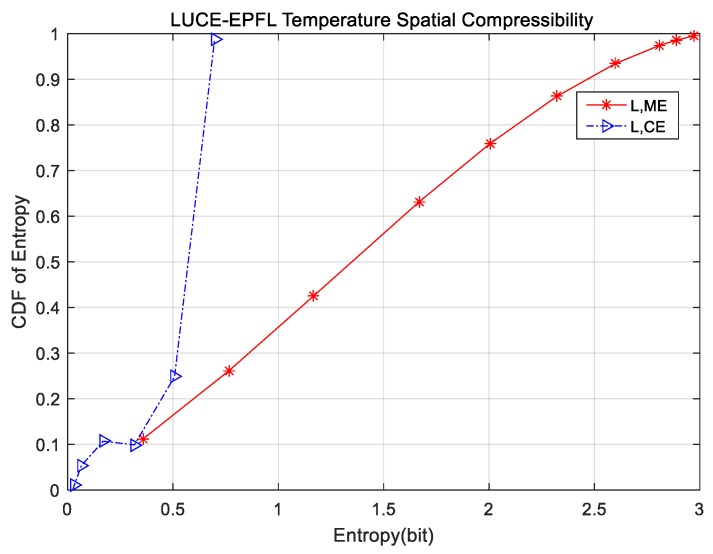
Spatial compressibility of LUCE-EPFL temperature.

**Figure 2:**
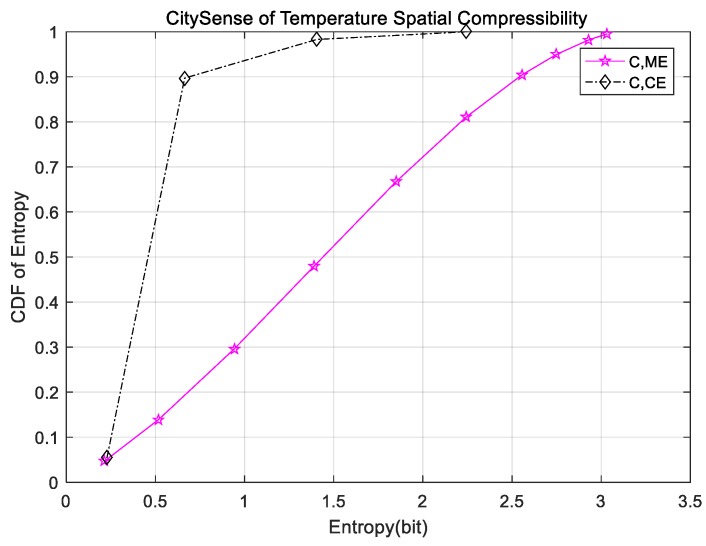
Spatial compressibility of CitySense temperature.

In this section, we pick up two temperature subsets from LUCE-EPFL and CitySense that are representative of the other datasets to approximately estimate spatial marginal and conditional entropy. The cumulative distribution functions (CDF) are provided in Figure 1 and Figure 2. In Figure 1, the red star curve represents CDF of marginal entropy, while the blue triangle curve is CDF of conditional entropy on LUCE-EPFL datasets. As can be seen in Figure 1, the values of marginal and conditional entropy are less than 3 bits. It is worth noting that conditional entropy is only about 0.7 bit. There is a big gap between the CDF curve of conditional entropy and the CDF curve of marginal entropy, which demonstrates that data storage space can be compressed considerably. Similarly, in Figure 2, the CDF of conditional entropy from CitySense is much smaller than that of marginal entropy. The same phenomenon with CitySense indicates the spatial compressibility of the sensor nodes’ readings in WSNs. We especially study five different datasets divided into classes based on the physical conditions and calculate the marginal entropy per signal. The results are recorded in Table 2. In Table 2, the first column indicates the marginal temperature entropy of different datasets. The entropy range is 2.5 bit to 3.1 bit. The other columns are humidity, solar radiation, wind, water, light, and voltage. The minimum marginal entropy is 0.592 bit of DEI light, while the maximum is 3.256 bit of CitySense wind in general. Therefore, it is inadvisable to store sensor node readings with 32 bit or 64 bit, that is to say, sensor node readings are compressible to some extent.

### 4.2. Modified Diffusion Wavelets

Conventional CS-based data-gathering approaches generally assume that sensor node readings have perfect sparse features under FFT, DWT, DCT, etc. To make full use of the spatial correlation property, which is demonstrated by experiments in Section 4.1, we take diffusion wavelets [36] as the sparse basis considering the spatial correlation of sensor node readings in WSNs. One is the nodes degree, and the other is the distance between the different sensor nodes. In addition, an improved QR decomposition of Givens transform is introduced to set up the sparse basis. Then, we describe how to construct the modified diffusion wavelets in detail. However, diffusion wavelets are affected significantly by the diffusion operator, which is equivalent to the wavelet function of a discrete wavelet transform. Diffusion is utilized as a smoothing and scaling technique to enable multi-scale and coarse-grained application. The detailed steps are shown in Algorithm 1.

**Figure 3:**
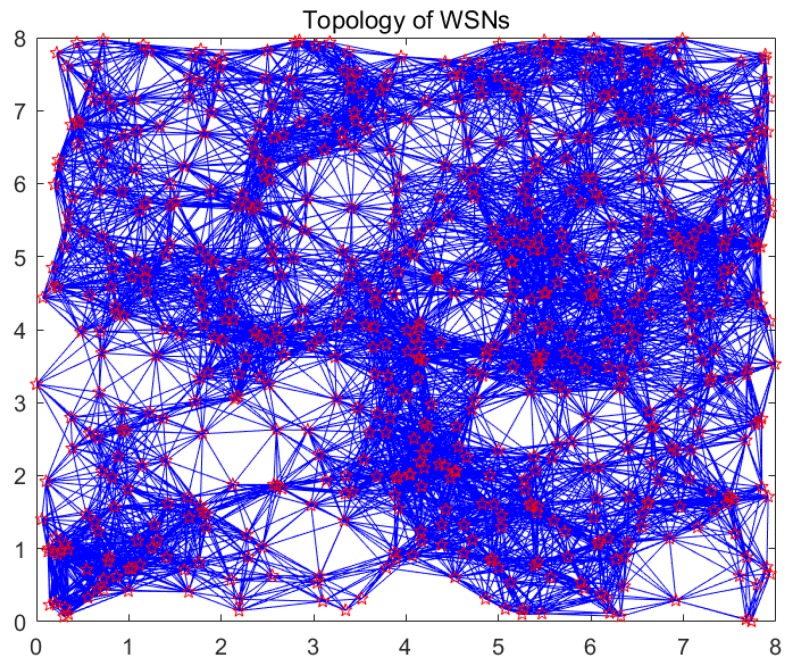
Topology of WSNs.

Step 1: Suppose that G(V,E) denotes a graph with N sensor nodes deployed in the monitoring environment, as indicated in Section 3.2. Diffusion wavelets are introduced to set up an orthonormal basis for functions supported by the topology graph of WSNs. In this section, we take a random deployment of WSNs to explain this process. WSNs’ topology of 640 sensor nodes is shown in Figure 3. In Figure 3, the hexagram vertex represents the sensor node, while the blue edge denotes the wireless link.

**Figure 4:**
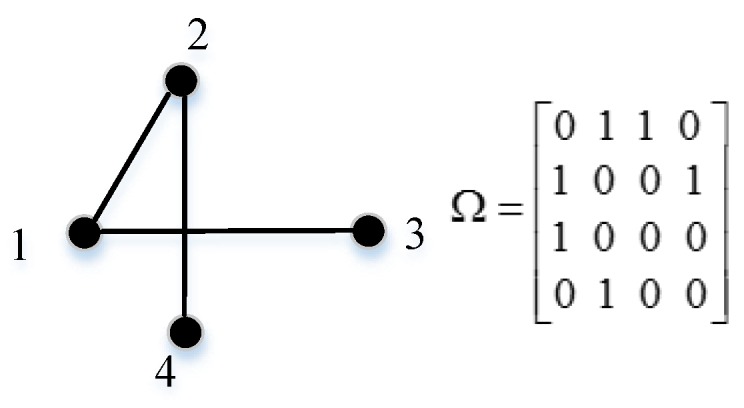
An example of the weighted adjacency matrix of a graph.

Step 2: Calculate the weight adjacency matrix of G(V,E), which is denoted as $\Omega \;=\;[w_{i,j}]$. $w_{i,j}$ is the weight of the edge in the graph. In this section, we consider two different cases of weight. The sensor node degree is chosen as the weight in the first scheme, while the distance between sensor nodes is taken into consideration to exploit the spatial correlation features, aiming to mitigate the load of WSNs in another scheme. In the former case, we show an example of a graph and corresponding weight adjacency matrix in Figure 4. In the latter case, the weight function is given below in Equation (11), which follows a similar method to [37].

(11) $$w_{ij}=\left\{ \begin{array}{ll} d_{ij}^{\gamma } & ,i\neq j,d_{ij}\leq r\\ \chi & ,\mathrm{otherwise} \end{array}\right.,$$

where r is the maximum distance among the sensor nodes that can directly communicate by a single hop. $d_{ij}$ is the Euclidean distance between node i and node j. γ is a negative number, while χ is a small positive number.

Step 3: Generate a normalized Laplacian matrix of G(V,E): $\Lambda =[\lambda_{ij}]$. In [38], Chung et al. indicates that Λ is the degree of correlations among different function values provided at the vertices of the graph G(V,E). In the first schedule, we denote $\lambda_{ij}$ using Equation (12), while the other schedule considering spatial correlation implements Equation (13). Generally speaking, an eigenvalue or eigenvector shows the special correlations at some scale. We need to split the space of Λ if we decompose the signal sampled of the G(V,E) in a multi-scale.

(12) $$\lambda_{ij}=\left\{ \begin{array}{ll} 1, & i=j\\ -\frac{w_{ij}}{\sqrt{\sum_{u}w_{iu}\sum_{u}w_{uj}}}, & \mathrm{otherwise} \end{array}\right.$$

(13) $$\lambda_{ij}=\left\{ \begin{array}{ll} 1-\frac{\chi }{\sum_{u}d_{iu}^{\gamma }}, & i=j\\ -\frac{d_{ij}^{\gamma }}{\sqrt{\sum_{u}d_{iu}^{\gamma }\sum_{u}d_{uj}^{\gamma }}}, & \mathrm{otherwise} \end{array}\right.$$

Step 4: However, the diffusion operator O stems from Λ, where O shares the same eigenvalues as Λ (less than 1). The diffusion operator is O=I−Λ or O=Λ/2; in this paper, we choose the first expression.

Step 5: Consequently, recursively raise O to power 2, and delete the diminishing eigenvalues with a threshold. Step by step, this approach splits the space spanned by the eigenvectors. Let the initial space of O be $x_{0}={\mathbb{R}}^{N}$, which is represented by scale space ${\text{\{}x_{j}\}}_{j\in N}$ and wavelet space ${\left\{V_{j}\right\}}_{j\in N}$. Wavelet space $V_{j}$ is different between $x_{j}$ and $x_{j+1}$. Then, we derive Equation (14):

(14) $$x_{j+1}=x_{0}⊕V_{0}⊕V_{1}⊕\cdots ⊕V_{j}.$$

Here, steps 5.1–5.5 accomplish the modified QR decomposition, where ${\left[O\right]}_{x_{a}}^{x_{b}}$ indicates the column space of matrix O denoted by basis $x_{b}$ at scale b, and row space is denoted by basis at scale a, ${\left[x_{b}\right]}_{x_{a}}$ represents basis $x_{b}$ denoted on the basis $x_{a}$.

Step 6: In the end, the diffusion wavelet basis Ψ is the concatenation of the scale functions and wavelet functions.

**Alogithm 1** Modified diffusion wavelets.**Input:** the number of sensor nodes N, communication radius r, decomposition level η, precision ε and MQR function.**Output:** sparse basis Ψ.1 generate a graph G(V,E)2 compute weight adjacency matrix $\Omega =[w_{i}{}_{j}]$ according to the vertex degree/Equation (11)3 calculate normalized Laplacian matrix Λ relying on Equation (12)/Equation (13)4 generate diffusion operator
O=I−Λ5 recursively raising Ο to power 2 5.1 **for**
η= 0 to η − 1 5.2 ${\left[x_{j+1}\right]}_{x_{j}}$, ${\left[O\right]}_{x_{0}}^{x_{1}}\leftarrow \mathrm{MQR}({\left[O^{2^{\eta }}\right]}_{x_{j}}^{x_{j}},\varepsilon )$ 5.3 $O_{j+1}:={\left[O^{2^{\eta +}{}^{1}}\right]}_{x_{j+}{}_{1}}^{x_{j+}{}_{1}}\leftarrow {\left[x_{j+1}\right]}_{x_{j}}{\left[O^{2^{\eta +}{}^{1}}\right]}_{x_{j}}^{x_{j}}{\left[x_{j+1}\right]}_{x_{j}}^{*}$ 5.4
${\left[\Psi_{\eta }\right]}_{x_{j}}\leftarrow \mathrm{MQR}(I_{\langle x_{j}\rangle }-{\left[x_{j+1}\right]}_{x_{j}}{\left[x_{j+1}\right]}_{x_{j}}^{*},\varepsilon )$ 5.5 **end for**6 concatenation of the scale functions and wavelet functions is regarded as the sparse basis Ψ.MQR Function:
Q,R←MQR(B,ε)**Input:**
*B*: N×N sparse matrix, ε**Output:**
Q, R matrix, possibly sparse, such that $B{=}_{\varepsilon }QR$(1) Q is orthogonal(2) R is upper triangular up to a permutation(3) The columns of Q
ε-span the space spanned by the columns of *B*

**Figure 5:**
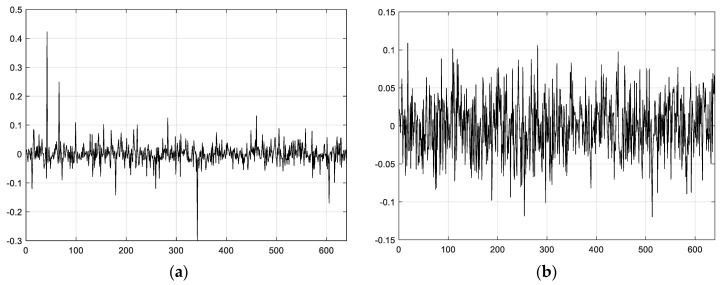
Wavelet functions of the first scheme: (**a**) 2nd-level wavelet function; (**b**) 10th-level wavelet function.

**Figure 6:**
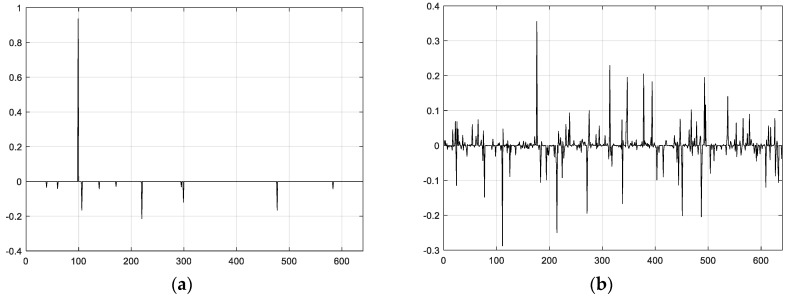
Wavelet functions of the second scheme: (**a**) First-level wavelet function; (**b**) 10th-level wavelet function.

Figure 3 denotes the topology of 640 nodes of WSNs, and also represents some scale functions. To visualize the wavelets’ function, we plot Figure 5 and Figure 6 using the first scheme (Equation (12)) and the second scheme (Equation (13)), respectively. Figure 5a introduces the second-level wavelet function, while Figure 5b is the 10th-level wavelet function for the former schedule. Figure 6 represents the second schedule considering the spatial correlation of sensor node readings in WSNs. Figure 6a,b indicate first- and 10th-level wavelet functions, respectively. Obviously, the second scheme is set up on a sparser basis, for it can capture the sensor node’s relationship and network topology properties and thus gain valuable information from the real world.

### 4.3. Modified Ant Colony Routing Algorithm

In this paper, in order to decrease the whole network transmission load and prolong the network lifetime, we provide a modified ant colony routing algorithm, where to speed up the convergence rate and avoid local optimal of the algorithm, pheromone impact factor is improved. Here, we select the energy consumption model described in Section 3.2. The traditional ant colony optimization algorithm selects the next hop depending on Equation (15) [39]:

(15) $$p_{ij}^{\vartheta }=\left\{ \begin{array}{ll} \frac{{\left[\tau_{ij}(t)\right]}^{\varsigma }{\left[\rho_{ij}(t)\right]}^{\xi }}{\sum_{\upsilon \subset allowed_{\vartheta }}{\left[\tau_{i\kappa }(t)\right]}^{\varsigma }{\left[\rho i_{j}(t)\right]}^{\xi }}, & j\subset allowed_{\vartheta }\\ 0, & others \end{array}\right.,$$

where $\tau_{ij}(t)$ denotes the pheromone information on edge (i,j), while $\rho_{ij}(t)$ is the heuristic information on edge (i,j). ς and ξ are impact factors demonstrating the importance degree of the pheromone information and heuristic information. In order to speed up the convergence rate and avoid local optimal, impact factor ς is modified as in Equation (16):

(16) $$\varsigma =\mu (1+e^{-10\times {\left(iter/totiter\right)}^{10}}),$$

where μ is a small positive constant ∈(0,1]; iter and totiter refer to current iterations and total iterations, respectively. In Equation (16), ς gradually becomes smaller as the number of iterations increases. In other words, the proportion of pheromones will diminish when the number of iterations rises.

Furthermore, to yield optimal routing by the ant colony algorithm, in this subsection, a sensor node’s residual energy and path length are taken into consideration simultaneously. So, the fitness value of each routing is presented as follows:

(17) $$Fitness=\beta (E_{ave}\times E_{\min})+\sigma Len_{\vartheta }^{-iter},$$

where $E_{ave}$ indicates the average residual energy, while $E_{\min}$ represents the node minimal energy of ants passing through the path. $Len_{\vartheta }^{-iter}$ denotes the reciprocal of path length for given ϑth ant and iterth iterations. β and σ are ∈[0,1] constants, and β+σ=1. Consequently, the path with the largest fitness function value is chosen as the optimal routing, thus balancing the network load and prolonging the network lifetime. Specifically, the modified ant colony algorithm is shown in Algorithm 2.

**Algorithm 2.**
Modified ant colony algorithm.**Input:**
the number of sensor nodes N, the power expended to run the transmitter or receiver circuitry of sensor node $E_{elec}$, energy consumption of multi-path fading amplifier $E_{amp}$, energy consumption of free-space amplifier $E_{fs}$, distance threshold $d_{0}$, impact factors of pheromone information ς, impact factors of heuristic information ξ, μ is a small positive constant ∈(0,1], pheromone information on edge (i,j)τ, heuristic information on edge (i,j)
ρ, β and σ are ∈[0,1] constants.**Output:** optimal routing Routing.1 Initialization routing R(Path,iter,ϑ), energy for each node and tabu2 calculate distance $d_{ij}$ of different nodes, $\rho_{ij}=1/d_{ij}$3 **while** maximum iterations has not be reached4  **for**
ϑ=1: $\Theta^{\prime }$5   compute $allowed_{\vartheta }$ according to the node communication radius.6   generate transition probability $p_{ij}^{\vartheta }$ based on Equations (15) and (16)7   choose the next hop node, relying on $p_{ij}^{\vartheta }$, modify routing and tabu8   the destination node or not? If not, go back to step 2, or proceed to step 99   update the node residual energy based on Equations (5) and (6), routing depending on Equation (17)10  **end for**11 **end while**12 return the optimal routing Routing.

### 4.4. Compressive Data Gathering

**Figure 7:**
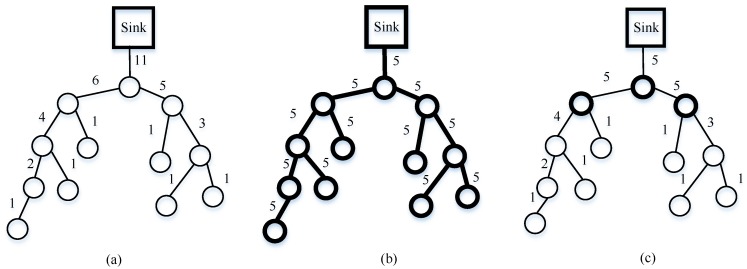
(**a**–**c**) Comparison of the three different data-gathering schemes.

**Figure 8:**
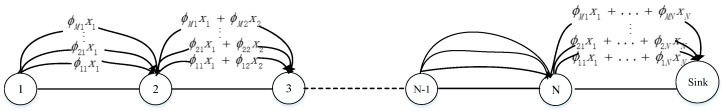
Compressive data gathering (CDG).

WSNs are utilized for gathering physical signal from the real world in practical applications. Without using CS theory, which is the simplest method, a data-gathering scheme with the help of the tree topology is shown in Figure 7a. In order to dramatically decrease communication costs and prolong the network lifetime, the authors of [12] consider that the sink node receives only M packets instead of N packets of original data from the whole network. In the end, at the sink, CS theory is used to reconstruct the original data. For the CDG algorithm, each node in the WSN multiplies its readings $x_{j}$ using the corresponding j column vector of basis matrix Φ. Next, the sensor node adds them to its own readings after receiving all same-size vectors from descendent nodes and transmitting the final results to its parent node with M packets. Let us illustrate the product of CDG in Figure 8, where Φ is M×N matrix, and each column corresponds to one weight sum. In the plain CS [11], all nodes in WSNs transmit M packets and each has equal transmission costs; therefore, each CS measurement cost remains relatively high. An example of the plain CS mechanism is given in Figure 7b. It is obvious that for these approaches (non-CS and plain CS), the former transmits fewer packets compared with plain CS from the point of view of child nodes. In [14], Wu et al. provides the hybrid CS method, where non-CS is chosen when the number of packets is less than or equal to M; alternatively, plain CS is used. Figure 7c illustrates the idea of hybrid CS. In Figure 7c, thin circles indicate a forward node using non-CS, while thick circles denote the gathering node using plain CS.

### 4.5. Data Gathering with Sparse Random Projections

**Figure 9:**
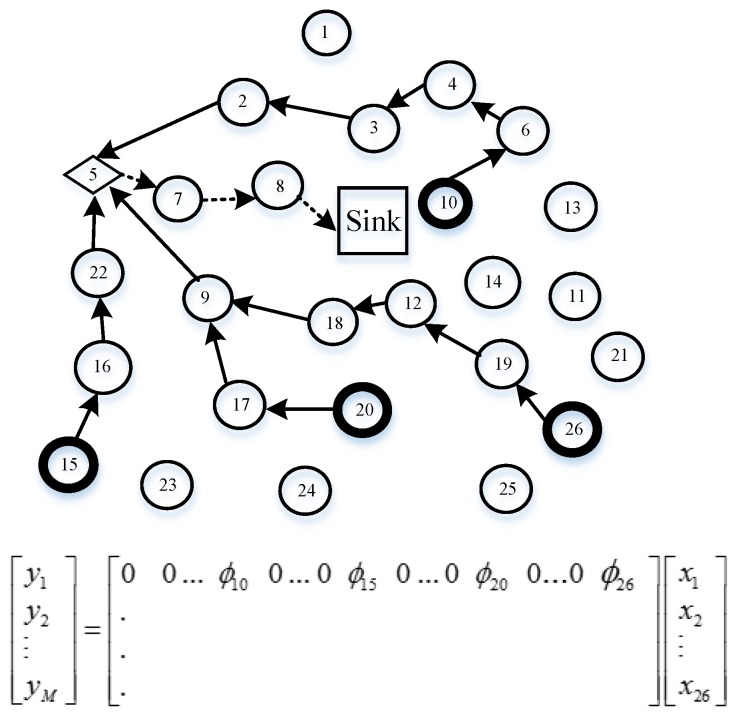
An example of data gathering per random projection node using Φ.

The aforementioned methods (plain CS and hybrid CS) still experience great challenges for all nodes involved in each measurement; in other words, the two mechanisms follow the dense matrix. Therefore, Wang et al. [23] propose a distributed data-gathering algorithm according to sparse random projections. In this algorithm, M nodes are randomly chosen to collect M weight sums in WSNs. Each projection sensor node collects one weight sum. Now, we explain how to accomplish data gathering in detail by means of Figure 9. Suppose that node 5 is a projection node, and $\phi_{ij}\neq 0$ of nodes 10, 15, 20, and 26. Then, node 5 initializes the projection by querying nodes 10, 15, 20, and 26. These nodes reply to node 5’s query with their readings $x_{j}$ in one packet. Finally, node 5 gathers all the data with its own data: $\sum_{j=1}^{N}\phi_{ij}x_{j}$ and transmits them to the sink node via the shortest path. Meanwhile, the transmission costs for each measurement reduce dramatically from O(N) in a dense matrix to O(logN) in a sparse random matrix.

### 4.6. A Novel Data-Gathering Scheme with Joint Routing and CS

Obviously, according to the analysis, the network load in hybrid CS is unbalanced. Specifically, sensor nodes near the sink node will consume more energy than those far from the sink node because of forwarding data more times. This results in sensor nodes near the sink dying earlier. However, [23] does not consider the total network costs for each random projection. To avoid the drawbacks, one can leverage the advantages of the algorithms; in this section, we present our data-gathering strategy combining joint routing and CS. 

Firstly, randomly choose M projection nodes in the network with probability $\frac{M}{N}$, which follows [23]. In the CS theory, the sink node needs M measurements to reconstruct the original data. Therefore, these M projection nodes will be selected as the gathering node, defined as $g_{1},g_{2},\ldots ,g_{m}$, to collect one random measurement $y_{i}$, and transmit $y_{i}$ to the sink node. Then, distribute non-zero elements in each row of measurement matrix Φ as uniformly as possible to guarantee the sparse features of the measurement matrix; the number of non-zero elements in each row should equal to $\left\lceil \frac{N}{M}\right\rceil$, which is related to Algorithm 3’s step 1. Additionally, each column of measurement matrix represents a sensor node, so if a column of the matrix has full zero elements, the data from its special sensor node should be thrown away. $\phi_{i}$, the column vector of measurement matrix Φ is required to store each sensor node memory in advance. Now, an example of measurement matrix is given in Equation (18), where M=5, and N=10.

Secondly, each row $\phi_{g_{1}}$ of measurement matrix Φ corresponds to one projection node. However, the number of each row coefficient is N, which is assigned to the size of network. $\phi_{i}{}_{j}\neq 0$ indicates that jth candidate nodes belong to the ith projection node’s. Here, this subsection corresponds to step 3 in Algorithm 3.

(18) $$\Phi =\left[\begin{array}{cccccccccc} 0 & 0 & 0 & \phi_{1,4} & 0 & \phi_{1,6} & 0 & 0 & 0 & 0\;\\ \phi_{2,1} & 0 & \phi_{2,3} & 0 & 0 & 0 & 0 & 0 & 0 & 0\\ 0 & 0 & 0 & 0 & \phi_{3,5} & 0 & 0 & \phi_{3,8} & 0 & 0\\ 0 & 0 & 0 & 0 & 0 & 0 & \phi_{4,7} & 0 & 0 & \phi_{4,10}\\ 0 & \phi_{5,2} & 0 & 0 & 0 & 0 & 0 & 0 & \phi_{5,9} & 0 \end{array}\right]$$

Subsequently, we set up the routing, which is from the offspring sensor nodes to the projection nodes and the projection nodes to the sink node, respectively. Based on the MST algorithm, access all candidate sensor nodes of a given projection node. In the first stage, the projection node is considered one root node tree. In the step 4 initialization stage in Algorithm 3, $Tree_{i}$ is assigned by *i*, and the temporary variable temp also yields i. Then steps 5–12 use the MST algorithm to construct the tree, adding the candidate nodes step by step. If temp is not empty, step 6 deletes the top node of the temp queue and puts its neighbor node in the Tree and temp. The next step is to delete them from $Can_{i}$ if they belong to $Can_{i}$. Note that these candidate nodes must be directly connected to the parent node by a single hop. If there are still some candidate nodes not involved in the tree, the Dijkstra algorithm [40] is proposed, aiming to find the shortest path from the residual nodes to the tree (steps 14–19), and we add the residual candidate nodes (steps 20–22).

Finally, this loop of 13–26 lines will repeat until $Can_{i}$ is empty. The modified ant colony routing technique is utilized to transmit packets of projection nodes to the sink node, namely step 27 of Algorithm 3. Consequently, Algorithm 3 terminates by generating the optimal routing between the projection nodes and the sink node, and an M routing tree from the projection nodes to their own candidate nodes. Our novel algorithm (Algorithm 3) is shown in more detail. The modified ant colony algorithm jointly considers the sensor node’s residual energy and the path length, which will not only balance the whole network load, avoiding nodes near the sink node dying earlier, but will prolong the network lifetime. In this way, the transmission costs should be greatly decreased compared to hybrid CS.

**Figure 10:**
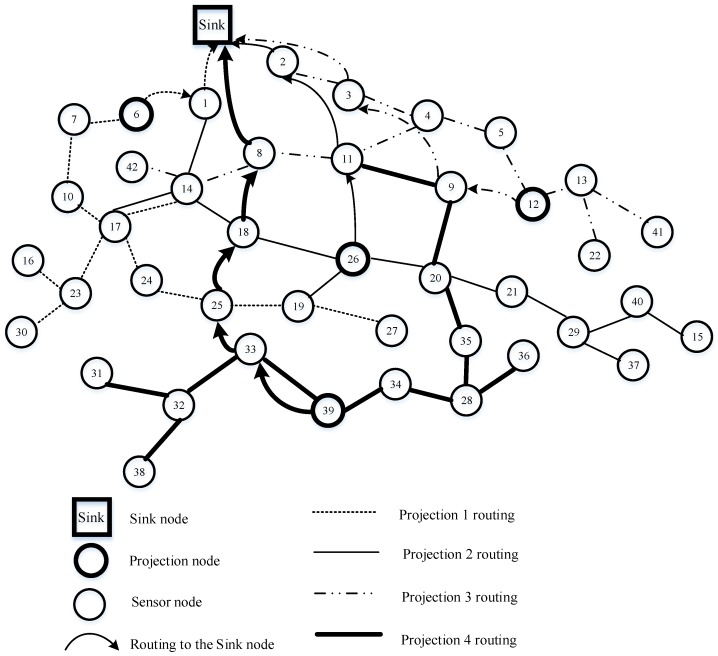
Our proposed algorithm, where N=42 and M=4.

Figure 10 is taken as an example to describe the details of Algorithm 3. It can be seen from Figure 10 that the thick circle indicates a projection node, the thin circle is a sensor node, the arc with an arrow represents the routing from the projection node to the sink node, and the dashed line, solid line, dotted and dashed line, and thick line describe four different projection routings. Sensor nodes 6, 12, 26, and 39 are chosen as projection nodes in the networks. Suppose that node 39 is the projection node in projection 4 routing, which has non-zero coefficients 9, 11, 20, 28, 31, 32, 33, 34, 35, 38, 39 of row vector $\phi_{4}$ in the measurement matrix. Here, projection node 39 wants to establish a tree using Algorithm 3. Above all, node 39 considers itself a tree with only one node. Select its neighbor nodes 33 and 34, which can be directly coupled with node 39 by a single hop in the following step. Next, node 33 retrieves candidate node 32 as its neighbor node, so node 32 is added to the tree, while node 34 finds node 28 is also its neighbor node; similarly, node 28 is involved in the tree. However, in the coming stage, there are no candidate nodes directly connected with the tree. Therefore, the Dijkstra algorithm is utilized to find the shortest path from the residual candidate nodes to the generated tree. Then nodes 31 and 38 are all joined to the tree, and nodes 35 and 36 are linked to node 28 by the shortest path. Again, nodes 20, 9, and 11 are involved in the tree using the MST algorithm. Finally, node 39 queries an optimal routing to the sink node for the projection node 39, which is 39→33→25→18→8→Sink, rather than 39→19→26→11→2→Sink using the Dijkstra algorithm. The reason is that the modified ant colony chooses the next hop considering not only residual energy but also path length. So, node 33 chooses node 25, which has more residual energy compared with node 19. The same reasoning is applied to node 25 and node 18. Node 1 near the sink node forwards data many times from the other nodes, leading to considerable energy reduction. Therefore, in order to ease the burden on the network, node 8 chooses a direct path to the sink node, instead of node 1, although node 1 is actually the shortest path. Similarly, the routing constructed in projections 1, 2, and 3 is shown in Figure 10. At the sink node, sensor signal reconstruction is implemented by Algorithm 4. In the process of the sensor signal, the gOMP [28] recovery algorithm is utilized, where $r_{it}$ denotes residual error, it is iteration. ∅ represents the null set, $a_{j}^{\prime }$ is jth of A. $\Lambda_{it}$ denotes the sets of indexes of it iteration.

**Algorithm 3.** Our proposed algorithm.**Input:**
G(V,E)**Output:**
Tree{1,2,…M}, Optr1 randomly select M sensor nodes $g_{1},g_{2},\ldots ,g_{M}$ in the network probability $\frac{M}{N}$, generate Φ2 **for**
i=1:M3   query candidate nodes $Can_{i}$($\phi_{i}{}_{j}\neq 0$) of projection nodes i4   initialization $Tree_{i}\leftarrow i$, $temp_{i}\leftarrow i$5   **while** !empty(temp) **do**6     CanNode←Del(temp)7     **if**
CanNode is i’s candidate node8      Tree←CanNode9      temp←CanNode10     $Del(Int_{i},CanNode)$11    **end if**12   **end while**13  **while** !empty($Can_{i}$) **do**14    **for** all residual candidate nodes $r\in Can_{i}$15    Path(r)← find a shortest path to $Tree_{i}$ using the Dijkstra algorithm16      **if**
shortestpath > Path(r)17         shortestpath←Path(r)18      **end if**19    **end for**20   Tree←shortestpath21   temp←shortestpath22   $Del(Can_{i},shortestpath)$23   **while** !empty(temp) **do**24   go back to steps 7–1125   **end while**26  **end while**27 Optr← Optimal routing from i to the sink node using Algorithm 228  return Tree{1,2,…M}29 **end for**

**Algorithm 4.**
Sensor signal reconstruction.1
**Input:** received data X, measurement matrix Φ, the number of atom is $n_{atom}$2 **Output:** reconstruct data $\overset{\wedge }{x}$3 generate sparse basis Ψ using Algorithm 14 collect data Y in the network using Algorithm 35 A←Ψ∗Φ6 initialization residual error $r_{0}=y$, $\Lambda_{0}=\emptyset$, $A_{0}=\emptyset$, it=17 compute $\langle r_{i-1},a_{j}^{\prime }\rangle$, select the largest la values from $\langle r_{i-1},a_{j}^{\prime }\rangle$; these values correspond to A’s column indexes j, constructing set $J_{0}$8 set $\Lambda_{it}=\Lambda_{it-1}\cup J_{0}$, $A_{it}=A_{it-1}\cup a_{j}$ (for all $j\in J_{0}$)9 ${\overset{\wedge }{x}}_{it}={\left(A_{it}^{T}A_{it}\right)}^{-1}A_{it}^{T}y$10 update $r_{it}=y-A_{it}{\overset{\wedge }{x}}_{it}$11 it=it+1, if it≤K go back to step 7, or proceed to step 1212 reconstruct $\overset{\wedge }{x}$, which is the generation value of the last iteration ${\overset{\wedge }{x}}_{it}$.

## 5. Theoretical Analysis

In this section, we prove that the equivalent sensing matrix A follows the RIP property. We first propose some definitions and corollaries.

**Definition** **2.** (*Sub-Gaussian [41]) A random variable* ℘ *is called sub-Gaussian if there exists a constant* c>0 *such that*

(19) $$E(\exp(℘\;t^{\prime }))\leq \exp(c^{2}{t^{\prime }}^{2}/2)$$

*holds for all* $t^{\prime }\in \mathbb{R}$. *We use the notation* $℘\sim Sub(c^{2})$ *to denote that* ℘ *satisfies Equation (19).*

**Theorem** **1.** *([41]) Suppose* ${℘}_{\partial }={\left[{℘}_{\partial 1},{℘}_{\partial 2},\ldots ,{℘}_{\partial M}\right]}^{\prime }$*, where each* ${℘}_{\partial i}$ *is i.i.d.* ${℘}_{\partial i}\sim Sub(c^{2})$ *and* $E({℘}_{\partial i}^{2})=\sigma^{2}$*. Then*

(20) $$E({\left\|{℘}_{\partial i}\right\|}_{2}^{2})=M\sigma^{2}$$

*Furthermore, for any* $\alpha^{\prime }\in (0,1)$, $\beta^{\prime }\in [c^{2}/\sigma^{2},\beta_{\max}]$*, there exists a constant* $c^{*}$ *such that*

(21) $$P({\left\|℘\right\|}_{2}^{2}\leq \alpha^{\prime }M\sigma^{2})\leq \exp(-M{\left(1-\alpha^{\prime }\right)}^{2}/c^{*})$$

*and*

(22) $$P({\left\|℘\right\|}_{2}^{2}\leq \beta^{\prime }M\sigma^{2})\leq \exp(-M{\left(\beta^{\prime }-1\right)}^{2}/c^{*})$$

**Lemma** **1.** *Fix* δ∈(0,1), A=ΨΦ *satisfies* $(1-\delta_{K}){\left\|x\right\|}_{2}^{2}\leq {\left\|\Phi x\right\|}_{2}^{2}\leq (1+\delta_{K}){\left\|x\right\|}_{2}^{2}$ *for all* N*-dimensional* K*-sparse signal* x.

**Proof.**  As mentioned above, diffusion operator O generated by Λ, while O has the same eigenvalues as Λ (less than 1). The modified diffusion wavelets are the concatenation of the scale functions and wavelet functions. Hence, we believe that the entries of the representative basis Ψ are randomly sequenced, which is denoted by ${℘}_{1},{℘}_{2},{℘}_{3},\ldots {℘}_{N}$. Moreover, since the non-zero entries for each row of measurement matrix Φ are mutually independent, the same probability is chosen for the projection nodes. Therefore, each row of A independently selects elements at random from Ψ. Alternatively, we suppose that A is generated by independent and identically distributed (i.i.d.)random variables ${℘}_{\partial }{}_{1},{℘}_{\partial 2},{℘}_{\partial 3},\ldots {℘}_{\partial M}$.

The next step is normalization, $A=\sqrt{\frac{N}{M}}{\left[\Theta_{1},\Theta_{2},\ldots ,\Theta_{M}\right]}^{T}$; in addition, we obtain Equations (23) and (24), which follow a similar idea as [18]:

(23) $$E(\Theta^{T}{\left(i,j\right)}^{2})={\left(\sqrt{\frac{N}{M}}\right)}^{2}E(\Theta {\left(j,i\right)}^{2})=\frac{1}{M}$$

(24) $$E(\Theta {\left(i,j\right)}^{T})=\sqrt{\frac{N}{M}}E(\Theta (j,i))=0.$$

Accordingly, we yield

(25) $$\begin{array}{l} E({\left\|Y\right\|}_{2}^{2})\\ =E(\sum_{i=1}^{M}{\left(<\sqrt{\frac{N}{M}}\Theta_{i},x>\right)}^{2})\\ =\sum_{i=1}^{M}E(\sum_{j=1}^{N}\left(\Theta^{T}(i,j\right)x_{j}){)}^{2}\\ =\sum_{i=1}^{M}\left(E(\Theta^{T}{\left(i,j\right)}^{2})\right){\left\|x\right\|}_{2}^{2}+2\sum_{j=1}^{N}\sum_{\kappa \neq j}E(\Theta^{T}(i,j))E(\Theta^{T}(i,j)x_{j}x_{\kappa }) \end{array}.$$

Here, we bring Equations (23) and (24) into Equation (25), and Equation (25) can be expressed as Equation (26):

(26) $$E({\left\|Y\right\|}_{2}^{2})={\left\|x\right\|}_{2}^{2}.$$

Alternatively, we set $\alpha^{\prime }=1-\delta$ and $\beta^{\prime }=1+\delta$. Hence, Equations (21) and (22) can be expressed as follows:

(27) $$P({\left\|Y\right\|}_{2}^{2}\leq (1-\delta )E({\left\|Y\right\|}_{2}^{2}))\leq \exp(-M\delta^{2}/c^{*})$$

(28) $$P({\left\|\Phi x\right\|}_{2}^{2}\leq (1-\delta ){\left\|x\right\|}_{2}^{2})\leq \exp(-M\delta^{2}/c^{*})$$

and

(29) $$P({\left\|\Phi x\right\|}_{2}^{2}\geq (1+\delta ){\left\|x\right\|}_{2}^{2})\leq \exp(-M\delta^{2}/c^{*}).$$

However, there are (N,K) possible K-dimensional subspaces of A relying on Sterling’s approximation. So, the inequality is given as $(N,K)\leq {\left(eN/K\right)}^{K}$. Thus, the probability of signal x needs to obey the conditions of Equation (30):

(30) $${\left(eN/K\right)}^{K}\cdot 2e^{\left(-M\delta^{2}/c^{*}\right)}=2e^{\left(-M\delta^{2}/c^{*}+K\log(N/K)+1\right)},$$

such that

(31) $$(1-\delta_{K}){\left\|x\right\|}_{2}^{2}\leq {\left\|\Phi x\right\|}_{2}^{2}\leq (1+\delta_{K}){\left\|x\right\|}_{2}^{2}.$$

Finally, M=O(Klog(N/K)) is selected to follow the RIP property with the probability approximation to 1, which completes the proof. 

## 6. Simulation Results

**Figure 11:**
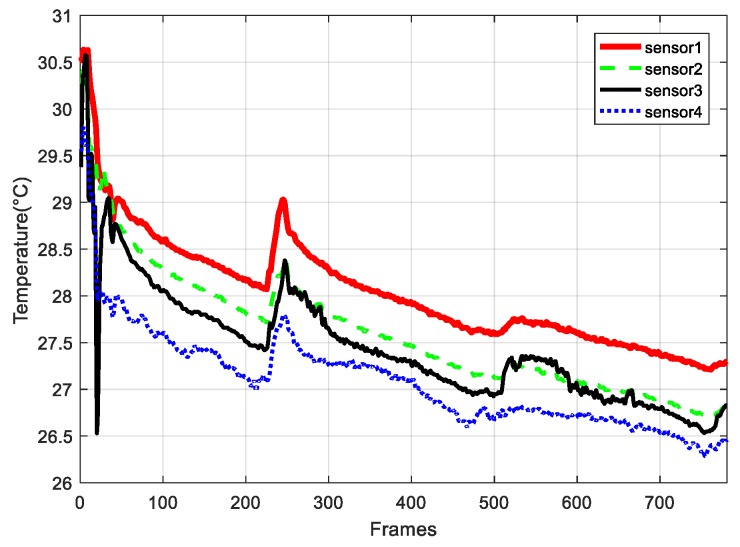
Original signal with different sensor nodes from DEI datasets.

In the following section, we evaluate the performance of our scheme by experiments. We choose the dataset from DEI [30], described in Section 4.1 and synthetic data. We evaluate our scheme mainly in terms of the sparse basis comparison; the reconstruction performance of the novel mechanism; the reconstruction error for different schemes; the energy consumption based on non-CS, plain CS, hybrid CS and our proposed algorithm (sparse basis is based on distance); and network lifetime performance between the different schemes and our algorithms. In our simulations, all programs have been run in the Matlab platform. Moreover, $E_{elec}=50\;\mathrm{nJ}/\mathrm{bit}$, $E_{amp}=60\;{\mathrm{pJ}/\mathrm{bit}/m}^{4}$, $E_{fs}=100\;{\mathrm{pJ}/\mathrm{bit}/m}^{2}$, L=1024 bits, initial energy $E_{0}=5\;J$. Figure 11 indicates the temperature signal from sensor nodes 1, 2, 3, and 4 in the detected area, and the frame length is 781. It is obvious that the signals have high spatial correlations, which is also demonstrated in Section 3.1.

### 6.1. Sparse Comparison

**Figure 12:**
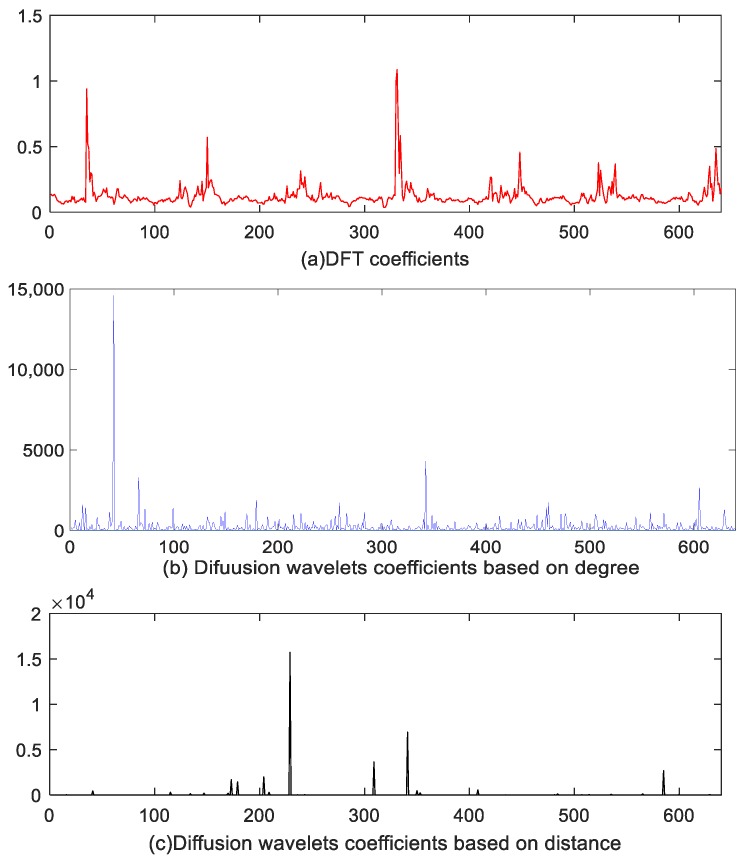
Comparison of different represent basis (**a**) DFT coefficients (**b**) coefficients based on degree (**c**) coefficients based on distance.

We compare the DFT sparse basis, diffusion wavelets based on degree (the first scheme), and diffusion wavelets based on distance (the second scheme) presented in Section 4.2. However, since there are not enough real data, we choose synthetic datasets [42] to accomplish the following simulations. Thus, Figure 12 is plotted in Matlab software. In Figure 12a denotes DFT coefficients, Figure 12b is the diffusion wavelet coefficients based on degree, and Figure 12c represents diffusion wavelet coefficients based on distance. Here, we select 650 frame length data. As can be seen from Figure 12, DFT does not sparisty the sensor signal, and the value of most of the coefficients is approximately 0.1 instead of 0. However, Figure 12b,c can all sparsity the sensory data, while the energy of the latter is more concentrated withoutinterference signal compared with the former. On the whole, diffusion wavelets based on distance represent better performance because they exploit the spatial correlation features among sensor nodes of the networks.

### 6.2. Performance of Reconstruction Signal

**Figure 13:**
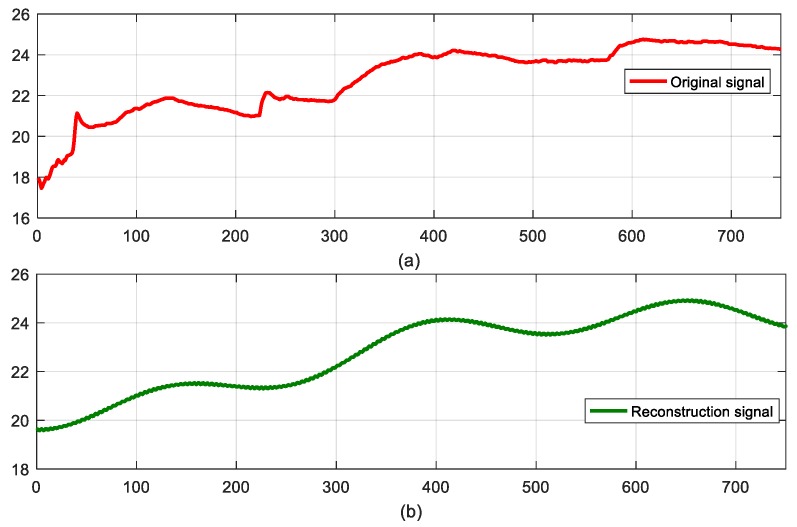
Comparison between original signal and reconstruction signal (**a**) original signal (**b**) reconstruction signal.

We implement our experiment using reconstruction algorithm gOMP. In our experiment, we choose modified diffusion wavelets based on distance and sparse matrix as our represent basis and measurement matrix, respectively. In order to show the recovery quality of the proposed algorithm, we plot Figure 13, which illustrates the error of the reconstruction signal decoding at the sink node. In our experiment, we define the reconstruction error as in Equation (32):

(32) $$\varepsilon_{1}={\left\|x-\overset{\wedge }{x}\right\|}_{2}\cdot {\left\|x\right\|}_{2}^{-1}.$$

We select 750 humidity readings, which are described in Figure 13a. Then, Figure 13b shows the recovery performance of sensor node readings with 256 CS measurements. This also demonstrates that 256 CS measurements can reconstruct the original signal with a relative recovery error of $\varepsilon_{1}=0.0306$.

### 6.3. Reconstruction Error for Different Schemes

**Figure 14:**
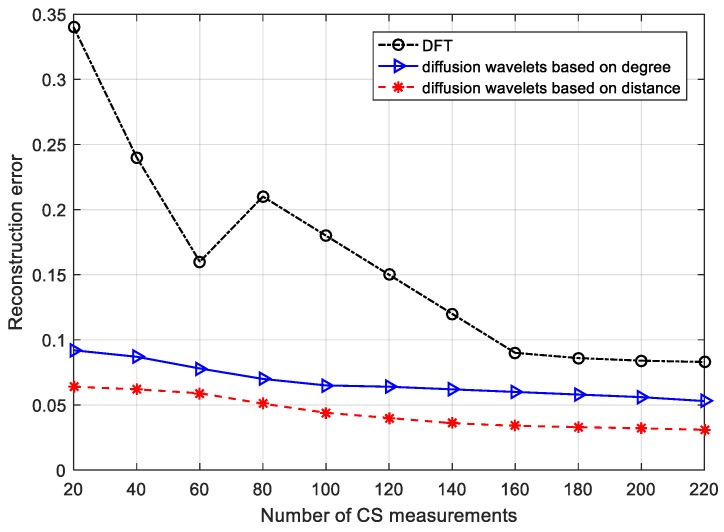
Reconstruction error vs. number of CS measurements.

Figure 14 compares the reconstruction error for DFT, diffusion wavelets based on degree, and diffusion wavelets based on distance. Here, we again extract the sensory data [42]. In Figure 14, the reconstruction error is high under DFT sparse basis, where the error is up to 0.34 when the number of CS measurements is 20. However, the recovery error is less than 0.1 when the number of CS measurements is 220. In other words, the method does not accurately recover the signal. The blue curve with triangles in Figure 14 indicates the reconstruction performance of diffusion wavelets based on degree. It is noted that the scheme can sparsity the signal, and can recover the original signal with smaller reconstruction error than the DFT at the sink node. The reconstruction error changes steadily along with the increase of the number of CS measurements. The reason is that the approach only considers some featured such as the network topology, namely the number of neighbor nodes for a given node, without capturing the geographical position features of the neighbor nodes. Accordingly, the diffusion wavelets based on distance take advantage of the favorable conditions to promote recovery, as is shown in the red star curve in Figure 14.

### 6.4. Energy Consumption Evaluation

**Figure 15:**
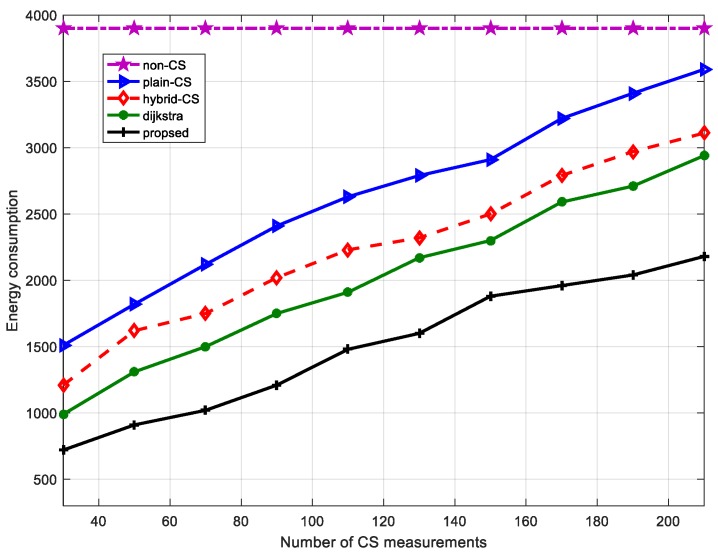
Energy consumption comparison of different data-gathering schemes with a change in the number of CS measurements.

To illustrate the efficiency of our proposed data-gathering technique, we compare the results obtained from the non-CS, plain CS, and hybrid CS data-gathering schemes for data to the sink node using Dijkstra algorithm and our proposed algorithm, where measurement M∈[30 220] and the frame length of data is 750. The number of sensor nodes is 80. From Figure 15, it can be seen that non-CS consumes the most energy, about $3.91\;\times \;10^{3}$ J, and the value is unchanged as the number of CS measurements steadily increases because the scheme does not adopt the CS technique. In addition, hybrid CS consumes about $1.22\;\times \;10^{3}$ J compared with $1.51\times 10^{3}$ J for plain CS when the number of CS measurements is 50. Both display an upward trend with the rise in the number of CS measurements, but the energy consumption of hybrid CS is always less than that of plain CS. The difference is about $0.46\;\times \;10^{3}$ J when the number of CS measurements is at its maximum, 220. The reason is that the hybrid CS scheme uses the CS mechanism or not based on the number of transmission packets. Therefore, hybrid CS takes advantage of non-CS and plain CS to reduce the energy consumption of the network. However, the data-gathering scheme using the Dijkstra algorithm consumes less energy than hybrid CS because the former makes full use of our modified diffusion wavelets and sparse random matrix. When the number of measurements is about 110, the gap between hybrid CS and Dijkstra reaches a maximum; when the number of CS measurements is 220, the difference is minimal. Our proposed data-gathering scheme using diffusion wavelets based on distance and a modified ant colony algorithm shows better performance compared with other methods. It is obvious that it considers a sensor node’s distance to exploit spatial correlation and sensor nodes’ residual energy and path length are jointly taken into consideration. Thus, the scheme consumes less energy. When the number of CS measurements achieves the maximum (220), the advantage of this approach is more prominent: the transmission cost is about $2.18\times 10^{3}$ J. This is far less than for the other four schemes in terms of energy consumption. From Figure 14 and Figure 15, we can observe that although the recovery error has become smaller, the transmission cost has increased a lot. Therefore, there is a trade-off between energy consumption and recovery error.

**Figure 16:**
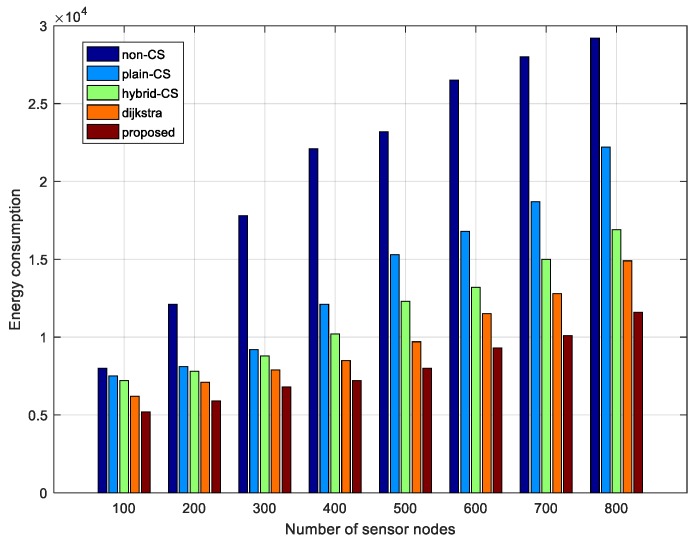
Energy consumption comparison of different data-gathering schemes vs. the number of sensor nodes.

Moreover, to further evaluate the performance of the proposed algorithm, Figure 16 is plotted, where energy consumption comparisons are shown among different data-gathering schemes with a change in the number of sensor nodes. It can be seen from Figure 16 that the non-CS technique always consumes the most energy as the number of sensor nodes increases from 100 to 800 because it does not use any compressive methods. However, plain CS dramatically reduces the network energy consumption compared with the non-CS. The reason is that it takes advantage of compressive sensing. Obviously, the energy consumption of hybrid CS decreases compared with plain CS. The number of sensor nodes is fewer than 400 and the difference between plain CS and hybrid CS is small; when the number of sensor nodes is 800, the gap between plain CS and hybrid CS is largest. That is to say, the energy efficiency of hybrid CS is more significant with an increase in the number of sensor nodes. Additionally, the transmission costs of the Dijkstra algorithm are lowered because of the sparse measurement matrix, as is shown in Figure 16. The performance of our proposed algorithm is better than all of the abovementioned schemes. Especially when the number of sensor nodes is greater than 500, the performance of our algorithm is more significant. This is why sparse represent basis is used and our improved ant colony algorithm is presented in the process of data gathering.

### 6.5. Network Lifetime Performance

**Figure 17:**
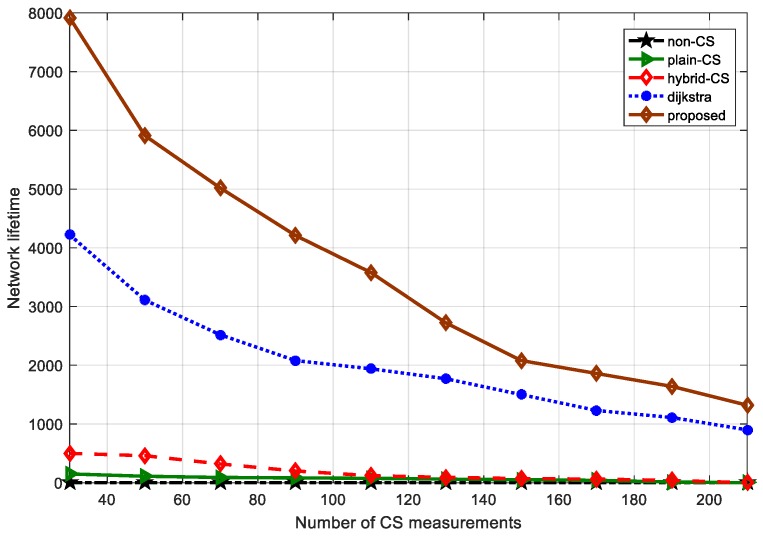
Network lifetime compassion of different data-gathering schemes vs. number of CS measurements.

**Figure 18:**
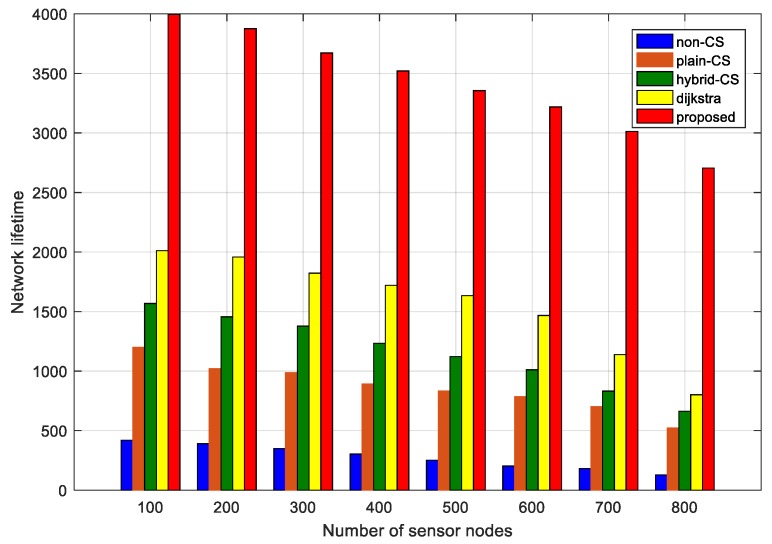
Network lifetime compassion of different data-gathering schemes vs. number of sensor nodes.

To complete the evaluation of the network lifetime performance, in this subsection we suppose that the time of the first dead node corresponds to the network lifetime. Figure 17 represents the relationship between the network lifetime and the number of CS measurements among non-CS, plain CS, hybrid CS, Dijkstra, and our proposed algorithm, while Figure 18 demonstrates the relationship between the network lifetime and the number of sensor nodes based on the different CS schemes and our algorithm. In Figure 17, we see that the network lifetime of the three data-gathering schemes non-CS, plain CS, and hybrid CS is short, and the number of CS measurements varies from 30 to 220. However, the network lifetime of Dijkstra and our algorithm is longer than in the above three schemes. The main reason is that the two methods also utilize a sparse measurement matrix. However, the advantage of our proposed algorithm is that the projection sensor nodes select the optimal routing to the sink node, aiming to lessen the transport load of the nodes nearest the sink node. Furthermore, the improved ant colony algorithm considers the residual energy of the sensor nodes, guaranteeing the load balance of the whole network, as is observed in Figure 17. We plot Figure 18 comparing the network lifetime of the different schemes with a change in the number of sensor nodes. It is obvious that our proposed algorithm is better than the other techniques. In Figure 18, we see that the red bar denotes the network lifetime of our algorithm, which is greater than that found with the other algorithms. When the number of sensor nodes is 800, in terms of the network lifetime, non-CS is about 200, plain CS is about 500, hybrid CS is about 620, Dijkstra is about 850 and our proposed algorithm is about 2600, respectively. Even though the number of sensor nodes is small, ~100–200, the network lifetime of the other schemes is far shorter than that of our proposed algorithm.

## 7. Conclusions and Future Work

Conventional CS data-gathering schemes design the sparse represent basis such as DCT, DFT, and wavelets transform do not consider the network topology and sensor nodes’ spatial information. Therefore, in our mechanism, diffusion wavelets based on sensor nodes’ degree and different nodes’ distance considering the above factors are proposed. Additionally, to further reduce the transport costs in WSNs, a sparse measurement matrix is utilized and MST and modified ant colony routing are jointly applied to mitigate energy consumption and balance the network load, especially lowering the transmission costs for those nodes nearest the sink node. Experimental results have shown that our sparse basis can sparsity the signal well. Our methods can also accurately reconstruct the original signal. Moreover, the reconstruction error of our scheme is less than DFT. Compared with existing data-gathering approaches, our proposed algorithm not only minimizes the energy consumption of the network, but prolongs the network lifetime. Furthermore, our algorithm has a theoretical guarantee of recovering the original signal, and the sink node needs to gather M = O(Klog(M/K)) measurements so as to recover the original signal. 

Future work should consider the following aspects: On the one hand, sensor node readings have not only spatial correlation, but temporal correlation, so our future work will extend the spatial–temporal filed. On the other hand, [43] demonstrates that optimization projection will generate better recovery performance than random projection, so a possible extension to this work will consider how to design the optimization projection so as to reduce the energy consumption.

## Figures and Tables

**Table 1:** Details of datasets in WSNs.

Name	Time Period	Physical Signal	Matrix Size	Frame Length
LUCE-EPFL	12–15 January 2007	Temperature, Humidity, Solar Radiation, Wind, Water	81 nodes × 856	5 min
IntelLab	28 February 2004–5 April 2004	Temperature, Humidity, Light, Voltage	54 nodes × 500	30 s
CitySense	14 October 2009–21 November 2009	Temperature, Wind	8 nodes × 887	60 min
DEI	19–22 March 2009	Temperature, Humidity, Light	29 nodes × 781	5 min
OrangeLab	26–27 August 2008	Temperature, Light, Voltage	75 nodes × 65	15 min

**Table 2:** Analysis of the marginal entropy (in bits) of different datasets in WSNs.

Name	Temperature	Humidity	Solar Radiation	Wind	Water	Light	Voltage
LUCE-EPFL	2.971	2.810	2.450	2.484	2.991	—	—
IntelLab	2.543	1.629	—	—	—	2.151	1.015
CitySense	3.034	—	—	3.256	—	—	—
DEI	2.589	2.510	—	—	—	0.592	—
OrangeLab	2.832	—	—	—	—	1.193	1.836
